# Association Between Relative Fat Mass and the Serum Creatinine/Cystatin C Ratio and Cardiometabolic Multimorbidity: Evidence From Two Large Population‐Based Surveys

**DOI:** 10.1155/jdr/4075738

**Published:** 2025-12-22

**Authors:** Yanfen Hu, Lingxia Li, Ling Yan, Xiaoting Zhao, Qinfan Wang, Zhenjie Xu

**Affiliations:** ^1^ Department of Geriatric Endocrinology, Metabolism and Respiratory (the Cadre Ward), The Second Affiliated Hospital of Xi’an Jiaotong University, Xi’an, Shaanxi, China, xjtu.edu.cn; ^2^ Institute of Jianjiyue Biomedical Research Center, Xi’an, Shaanxi, China

**Keywords:** cardiometabolic multimorbidity, CHARLS, NHANES, relative fat mass, serum creatinine–cystatin C ratio

## Abstract

**Background:**

The serum creatinine–cystatin C ratio (SCR/CysC) and relative fat mass (RFM) are both important indicators reflecting muscle and fat content, respectively, and are closely related to metabolic diseases and cardiovascular diseases. However, the roles of SCR/CysC and RFM in cardiometabolic multimorbidity (CMM) remain unclear. This study is aimed at exploring the relationships between SCR/CysC, RFM, and CMM, providing a new perspective for the early identification and intervention of CMM.

**Methods:**

This study included 9292 Chinese participants from the CHARLS database and 3822 American individuals from the NHANES database. Multivariate logistic regression analysis, restricted cubic spline (RCS) plots, and subgroup analyses were employed to explore the associations of SCR/CysC and RFM with CMM.

**Results:**

Analyses based on two databases revealed that the level of SCR/CysC levels was significantly negatively correlated with the risk of CMM. Conversely, higher RFM levels were positively correlated with an increased risk of CMM. Specifically, compared to the lower quartile (Q1) of SCR/CysC, the highest quartile (Q4) was associated with a decreased risk of CMM (CHARLS: odds ratio (OR) = 0.89, 95% confidence interval (CI): 0.87–0.91, *p* < 0.001; NHANES: OR = 0.90, 95% CI: 0.86–0.94, *p* < 0.001). Compared to the lower quartile (Q1) of RFM, the highest quartile (Q4) was associated with an increased risk of CMM (CHARLS: OR = 1.18, 95% CI: 1.16–1.21, *p* < 0.001; NHANES: OR = 1.20, 95% CI: 1.15–1.25, *p* < 0.001). The RCS plot results further supported this relationship, demonstrating that after adjusting for multiple confounding factors, a decrease in SCR/CysC and an increase in RFM were both associated with a higher risk of CMM.

**Conclusions:**

This study found that SCR/CysC levels were negatively correlated with CMM risk, whereas RFM levels showed a positive correlation. Thus, SCR/CysC and RFM may both serve as potential biomarkers for CMM screening.

## 1. Introduction

Cardiovascular and metabolic comorbidities, also known as cardiometabolic multimorbidity (CMM), refer to the co‐occurrence of two or more cardiovascular or metabolic disorders in an individual, typically including hypertension, diabetes, coronary heart disease, or stroke [1, 2]. A large‐scale study of 500,000 Chinese adults found that CMM is the most prevalent multimorbidity pattern, affecting 6% of participants, which is higher than other patterns [3]. Importantly, individuals with CMM exhibit a shorter lifespan compared to those with only a single cardiometabolic disease [4]. Therefore, the early identification of CMM and the implementation of targeted intervention strategies are of great significance.

Obesity is a significant risk factor for CMM [5]. Traditional indicators such as body mass index (BMI), waist circumference (WC), and waist‐to‐height ratio (WHtR) can predict CMM risk but have limitations, particularly in accurately assessing obesity across different body types and fat distributions [6, 7]. While WC and WHtR are better predictors of cardiovascular disease (CVD) than BMI, they primarily reflect central adiposity [8]. The triglyceride–glucose waist circumference (TyG‐WC) index, which combines central obesity and lipid accumulation, shows high predictive accuracy for CVD but requires invasive blood measurements [9]. In contrast, a novel index relative fat mass (RFM), which considers height, WC, and sex, has demonstrated equal or superior predictive value for hypertension, metabolic syndrome, and CVD compared with BMI, WC, and TyG‐WC, without the need for invasive tests [10–13]. Although RFM is associated with various diseases [14], its specific link to CMM is still unclear.

There is a strong correlation between fat content and muscle mass. Previous studies have defined sarcopenic obesity by combining grip strength and BMI and found it positively correlated with CMM in middle‐aged and elderly populations [1]. Another study, using the 2019 Asian criteria for sarcopenia, also showed that sarcopenia in middle‐aged and elderly individuals is associated with an increased risk of CMM [15]. However, the assessment indicators for sarcopenia, such as the simple physical performance battery and appendicular skeletal muscle mass measurement, have limitations as they are time‐consuming or require specialized equipment for measurement. To address this, the current study introduces a new alternative indicator: the serum creatinine to cystatin C ratio (SCR/CysC) [16]. Creatinine, a byproduct of muscle metabolism primarily produced by skeletal muscle, has serum levels that correlate positively with muscle mass [17, 18]. Cystatin C, a small protein produced by nucleated cells and filtered by the kidneys, has relatively stable serum levels largely independent of muscle mass [16]. By calculating the SCR/CysC ratio, the influence of kidney function can be effectively minimized, allowing for a more accurate reflection of muscle mass status. Compared with traditional assessment methods, SCR/CysC is simpler and more convenient to obtain. However, its specific relationship with CMM requires further investigation.

Given the significant associations of both body fat content and muscle mass with CMM, this cross‐sectional study was aimed at examining the relationships between SCR/CysC, RFM, and CMM.

## 2. Materials and Methods

### 2.1. Study Populations

This study utilized two nationally representative databases: the China Health and Retirement Longitudinal Study (CHARLS) and the National Health and Nutrition Examination Survey (NHANES). CHARLS is a longitudinal survey targeting individuals aged 45 years and above in China, and data for this study were derived from its 2014–2015 wave. NHANES is a series of cross‐sectional surveys of the noninstitutionalized population in the United States, covering a wide range of population characteristics and health indicators. The 1999–2004 cycles of NHANES were selected for this study because CysC was measured only during this period. CysC is a key indicator for assessing renal function and muscle mass, and its availability is essential for calculating SCR/CysC.

In the CHARLS database, participants were excluded if they were aged below 45 years; did not have complete data for SCR, cystatin C, RFM, or CMM assessment; or had extreme BMI values. After these exclusions, a total of 9292 participants were included in the study. Similarly, in the NHANES database, participants aged below 45 years or without complete data for SCR, cystatin C, RFM, and CMM assessment were excluded, resulting in a final sample of 3822 participants.

The CHARLS survey received approval from the Biomedical Ethics Committee of Peking University, while the NHANES research protocol secured approval from the Research Ethics Review Committee of the National Health and Wellness Commission. Written informed consent was obtained from all participants.

### 2.2. Detection Method and Definition of Exposure Variables

Height and WC were measured in accordance with established standards and are reported in centimeters. The formula for calculating the RFM is as follows: 64 − (20 × height/WC) + (12 × gender), where “1” represents women and “0” represents men [19]. Serum creatinine levels were determined via the kinetic alkaline picrate method, also known as the Jaffe reaction technique. The serum creatinine values from 1999 to 2000 needed to be corrected by Deming regression according to the NHANES guidelines. The raw creatinine value obtained directly from NHANES 1999–2000, calculated using the formula where standard creatinine = 1.013 × original creatinine + 0.147, was employed, but correction was not required for NHANES 2001–2002 and 2003–2004 [20]. Serum CysC levels were determined via a CysC immunoassay provided by Siemens Healthcare Diagnostics. The minimum detectable concentration of CysC was 0.23 mg/L. In cases where the measured CysC level fell beneath this minimum detectable concentration, the reported value was represented by dividing the minimum detectable concentration by the square root of 2, resulting in 0.16 mg/L. The SCR/CysC ratio can be calculated by dividing the SCR value (expressed in milligrams/deciliter) by the CysC value (expressed in milligrams/liter) and then multiplying the result by 10 [21].

### 2.3. Outcome Variables

CMM denotes an individual who has been diagnosed with two or more cardiovascular or metabolic disorders, encompassing hypertension, diabetes, coronary heart disease, and stroke [1, 22]. The scoring criteria for related diseases are as follows:

1. Diagnostic criteria for hypertension: (a) Systolic blood pressure (SBP) > 140 mmHg or diastolic blood pressure (DBP) > 90 mmHg. (b) The doctor informed that he/she has hypertension. (c) Currently taking blood pressure‐lowering drugs. Meeting any of the above items can be considered as hypertension.
2. Diagnostic criteria for diabetes: (a) Fasting blood glucose (FBG) > 126 mg/dL, or glycated hemoglobin (HbA1c) ≥ 6.5*%*. (b) The doctor informed that he/she has diabetes. (c) Currently using any hypoglycemic drugs. Meeting one of the above items can be considered diabetes.
3. Diagnostic criteria for coronary heart disease: The doctor once informed that there was angina pectoris, coronary heart disease, or heart disease.
4. Diagnostic criteria for stroke: The doctor once informed that there was a history of stroke.

### 2.4. Covariates

The covariates include age, race, gender, educational level, marital status, smoking status, and drinking status. To ensure the consistency between the CHARLS and NHANES databases, participants over 45 years old were all screened as the research subjects. Marital status was classified into three categories: never married, married/living with a partner, or separated/divorced/widowed. The educational attainment was classified into two categories: below high school (LTHS) or high school or above (HS/HS+). Smoking and drinking were categorized as “yes” or “no” based on self‐reports. The BMI values were calculated based on height and weight in the CHARLS database or obtained directly from the NHANES database.

### 2.5. Statistical Analysis

All statistical analyses fully account for the complex sampling weights of NHANES and CHARLS. NHANES 1999–2004 comprises three biennial cycles, which are combined using the official 6‐year composite weight WTINT6YR (=WTINT2YR/3), specifying the design with the stratification variable SDMVSTRA and primary sampling unit SDMVPSU. CHARLS 2014–2015 adopts cross‐sectional individual weights r_weight and is combined with the stratification variable strata and the PSU unit to define the complex survey structure. Baseline characteristics were analyzed according to whether participants were defined as CMM. Continuous variables were expressed as mean ± standard deviation (mean ± SD), and categorical variables were expressed as counts and percentages (%). Analysis of variance (ANOVA) and chi‐squared statistical tests were used for analysis.

Multivariate logistic regression models were employed to verify the associations between RFM, SCR/CysC, and CMM. The outcomes were expressed in terms of odds ratios (ORs) with corresponding 95% confidence intervals (CIs). RFM and SCR/CysC were divided into quartiles, and each quartile was analyzed as a continuous variable to evaluate the linear trend between the quartiles. Model 1 was a rough model without any adjustments. Model 2 was adjusted according to age and gender. Model 3 was adjusted for age, gender, marital status, educational level, and smoking and drinking conditions. Nonlinear effects were modeled via restricted cubic spline (RCS) plots. Furthermore, possible interactions among the variables were explored, with adjustments made for age, race, gender, educational attainment, marital status, and smoking and drinking habits. In addition to the cross‐sectional analysis of correlations, a prospective survival analysis was further conducted using the follow‐up data from NHANES to evaluate the predictive value of RFM and SCR/CysC levels for the all‐cause mortality risk in CMM patients. All the statistical analyses were conducted via R statistical software (Version 4.2.2). *p* values less than 0.05 (*p* < 0.05) were considered statistically significant.

## 3. Results

### 3.1. Baseline Characteristics of the Study Participants

A total of 9292 individuals from the CHARLS database (Figure 1a) and 3822 from the NHANES database were enrolled in this study (Figure 1b). The sociodemographic characteristics of the CHARLS individuals are detailed in Table 1, revealing that 53.4% were female and 46.6% were male. This study revealed that the average age of all CHARLS participants was 62.4 ± 8.9 years old, with CMM patients having an average age of 64.9 ± 8.7 years old (comprising 41.7% male and 58.3% female). There were statistically significant differences in age, gender, marital status, drinking, BMI, SCR/CysC, WC, and RFM between CMM patients and non‐CMM participants (*p* < 0.001). Table 2 presents the sociodemographic characteristics of the NHANES participants, which included a subtotal of 3822 participants (48.7% male and 51.3% female). This study found that the average age of all NHANES participants was 59.7 ± 11.0 years old, whereas CMM patients had a mean age of 64.5 ± 10.8 years old (50.7% male and 49.3% female). Significant differences were observed between CMM and non‐CMM participants in terms of age, race, education, marital status, smoking, drinking, BMI, SCR/CysC, WC, and RFM (*p* < 0.001). In addition, there were significant differences between participants excluded due to missing data and those finally included in the study in terms of age, gender, marital status, education level, smoking and drinking habits, RFM, SCR/CysC, hypertension, diabetes, and CMM (Table S1).

Figure 1Flowchart of the participants′ selection process. (a) CHARLS 2015–2016 survey data. (b) NHANES 1999–2004 survey data.

**Figure:**
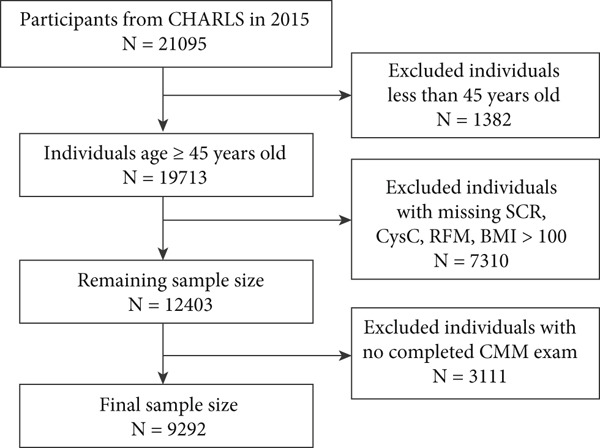
(a)

**Figure:**
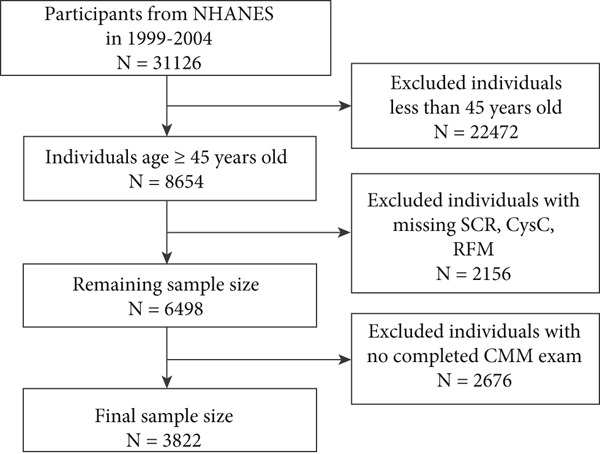
(b)

**Table 1:** Sociodemographic characteristics of CHARLS, 2015–2016.

**Characteristic**	**Overall**	**Non-CMM**	**CMM**	**p** **value**
Age (mean ± SD)	62.4 ± 8.9	61.9 ± 8.9	64.9 ± 8.7	< 0.001
Gender (%)				< 0.001
Female	4959 (53.4)	4066 (52.4)	893 (58.3)	
Male	4333 (46.6)	3693 (47.6)	640 (41.7)	
Marital status (%)				< 0.001
Married/living with a partner	7967 (85.7)	6708 (86.5)	1259 (82.1)	
Never married	63 (0.7)	56 (0.7)	7 (0.5)	
Widowed/divorced/separated	1262 (13.6)	995 (12.8)	267 (17.4)	
Education (%)				0.447
HS/HS+	924 (10.0)	763 (9.8)	161 (10.5)	
LTHS	8361 (90.0)	6991 (90.2)	1370 (89.5)	
Smoking (%)				0.073
No	5358 (57.7)	4442 (57.3)	916 (59.8)	
Yes	3929 (42.3)	3313 (42.7)	616 (40.2)	
Drinking (%)				< 0.001
No	6117 (65.9)	4989 (64.4)	1128 (73.6)	
Yes	3165 (34.1)	2761 (35.6)	404 (26.4)	
Height (mean ± SD)	157.7 ± 8.6	157.8 ± 8.5	157.5 ± 8.9	0.187
Weight (mean ± SD)	59.6 ± 11.8	58.8 ± 11.4	63.7 ± 12.7	< 0.001
BMI (mean ± SD)	23.9 ± 4.1	23.6 ± 4.0	25.6 ± 4.5	< 0.001
SCR (mean ± SD)	0.8 ± 0.3	0.8 ± 0.3	0.8 ± 0.3	0.005
CysC (mean ± SD)	0.9 ± 0.2	0.9 ± 0.2	0.9 ± 0.3	< 0.001
SCR/CysC (mean ± SD)	9.5 ± 2.7	9.6 ± 2.7	9.0 ± 2.4	< 0.001
WC (mean ± SD)	85.4 ± 13.2	84.3 ± 12.9	90.8 ± 13.3	< 0.001
RFM (mean ± SD)	31.9 ± 14.7	31.3 ± 14.6	34.9 ± 15.1	< 0.001

*Note:* The mean ± SD was used for continuous variables, and the percentage (%) was used for categorical variables.

Abbreviations: BMI, body mass index; CysC, cystatin C; HS/HS+, educational level of high school and above; LTHS, educational level below upper secondary; RFM: relative fat mass; SCR, serum creatinine; WC, waist circumference.

**Table 2:** Sociodemographic characteristics of NHANES, 1999–2004.

**Characteristic**	**Overall**	**Non-CMM**	**CMM**	**p** **value**
Age (mean ± SD)	59.7 ± 11.0	58.0 ± 10.5	64.5 ± 10.8	< 0.001
Gender (%)				0.200
Female	1883 (51.3)	1281 (52.0)	602 (49.3)	
Male	1939 (48.7)	1258 (48.0)	681 (50.7)	
Race (%)				< 0.001
Mexican American	865 (4.6)	552 (4.5)	313 (5.1)	
Other Hispanic	634 (8.7)	359 (7.1)	275 (12.9)	
Non‐Hispanic white	2070 (77.9)	1459 (80.0)	611 (71.9)	
Non‐Hispanic black	139 (4.2)	97 (4.2)	42 (4.3)	
Other race	114 (4.6)	72 (4.2)	42 (5.7)	
Marital status (%)				< 0.001
Married/living with a partner	2403 (70.7)	1639 (73.1)	764 (64.1)	
Never married	174 (4.6)	121 (4.4)	53 (4.9)	
Widowed/divorced/separated	1126 (24.7)	697 (22.5)	429 (30.9)	
Education (%)				< 0.001
Hs/Hs+	2364 (77.4)	1682 (81.1)	682 (66.8)	
LTHS	1457 (22.6)	856 (18.9)	601 (33.2)	
Smoking (%)				< 0.001
No	665 (34.7)	480 (37.1)	185 (28.1)	
Yes	1418 (65.3)	1602 (62.9)	1898 (71.9)	
Drinking (%)				< 0.001
No	1306 (32.6)	811 (29.8)	495 (40.4)	
Yes	2401 (67.4)	2896 (70.2)	3212 (59.6)	
Height (mean ± SD)	168.3 ± 10.0	168.7 ± 9.9	167.2 ± 10.1	< 0.001
BMI (mean ± SD)	28.9 ± 6.2	28.1 ± 5.6	31.2 ± 7.0	< 0.001
SCR (mean ± SD)	0.9 ± 0.5	0.9 ± 0.4	1.0 ± 0.6	< 0.001
CysC (mean ± SD)	0.9 ± 0.4	0.8 ± 0.3	1.0 ± 0.5	< 0.001
SCR/CysC (mean ± SD)	11.1 ± 3.4	11.3 ± 3.5	10.6 ± 2.9	< 0.001
WC (mean ± SD)	100.5 ± 15.3	98.1 ± 14.3	107.5 ± 15.9	< 0.001
RFM (mean ± SD)	36.0 ± 7.8	35.2 ± 7.6	38.2 ± 7.9	< 0.001

*Note:* The mean SD was used for continuous variables, and the percentage (%) was used for categorical variables.

Abbreviations: BMI, body mass index; CysC, cystatin C; HS/HS+, educational level of high school and above; LTHS, educational level below upper secondary; RFM, relative fat mass; SCR, serum creatinine; WC, waist circumference.

### 3.2. Association Between SCR/CysC, RFM, and CMM

Among CHARLS participants, the SCR/CysC level was negatively correlated with the risk of CMM, as shown in Table 3. There is a significant correlation between a higher SCR/CysC index and a lower incidence of CMM. After adjusting for potential confounding variables (Model 3), for every 1‐unit increase in SCR/CysC, the risk of CMM would decrease by 2% (OR = 0.98, 95% CI: 0.97–0.99). Similarly, the same conclusion was reached among NHANES participants, where SCR/CysC levels were negatively associated with the risk of CMM (Table 4).

**Table 3:** Multivariate logistic regression results of the relationships among SCR/CysC, RFM, and CMM in CHARLS.

**Parameters**	**Model 1**	**p** **value**	**Model 2**	**p** **value**	**Model 3**	**p** **value**
SCR/CysC index						
SCR/CysC_per_Q	0.97 (0.96,0.98)	< 0.001	0.98 (0.97,0.99)	< 0.001	0.98 (0.97,0.99)	< 0.001
Quartiles of SCR/CysC						
Q1	Ref		Ref		Ref	
Q2	0.93 (0.91, 0.95)	< 0.001	0.94 (0.92, 0.96)	< 0.001	0.94 (0.92, 0.96)	< 0.001
Q3	0.92 (0.90, 0.94)	< 0.001	0.94 (0.92, 0.96)	< 0.001	0.94 (0.92, 0.96)	< 0.001
Q4	0.89 (0.87, 0.91)	< 0.001	0.92 (0.90, 0.94)	< 0.001	0.92 (0.90, 0.95)	< 0.001
*p* for trend	< 0.001		< 0.001		< 0.001	
RFM index						
RFM_per_Q	1.04 (1.03, 1.04)	< 0.001	1.04 (1.03, 1.05)	< 0.001	1.04 (1.03, 1.05)	< 0.001
Quartiles of RFM						
Q1	Ref		Ref		Ref	
Q2	1.11 (1.09, 1.13)	< 0.001	1.13 (1.10, 1.15)	< 0.001	1.12 (1.10, 1.15)	< 0.001
Q3	1.05 (1.03, 1.08)	< 0.001	1.19 (1.15, 1.24)	< 0.001	1.19 (1.15, 1.24)	< 0.001
Q4	1.18 (1.16, 1.21)	< 0.001	1.34 (1.29, 1.39)	< 0.001	1.34 (1.29, 1.39)	< 0.001
*p* for trend	< 0.001		< 0.001		< 0.001	

*Note:* Model 1: no adjustment for any potential influencing factors. Model 2: adjusted for age and gender. Model 3: adjusted for age, gender, marital status, education, smoking, and drinking.

**Table 4:** Multivariate logistic regression results of the relationships between SCR/CysC or RFM and CMM in NHANES.

**Parameters**	**Model 1**	**p** **value**	**Model 2**	**p** **value**	**Model 3**	**p** **value**
SCR/CysC index						
SCR/CysC_per_Q	0.97 (0.96, 0.99)	< 0.001	0.98 (0.97, 1.00)	0.035	0.99 (0.97, 1.01)	0.371
Quartiles of SCR/CysC						
Q1	Ref		Ref		Ref	
Q2	0.90 (0.86, 0.94)	< 0.001	0.91 (0.87, 0.95)	< 0.001	0.88 (0.83, 0.94)	< 0.001
Q3	0.94 (0.90, 0.97)	0.002	0.95 (0.91, 0.99)	0.014	0.91 (0.86, 0.97)	0.005
Q4	0.90 (0.86, 0.94)	< 0.001	0.93 (0.89, 0.98)	0.006	0.93 (0.87, 0.99)	0.032
*p* for trend	< 0.001		< 0.001		< 0.001	
RFM index						
RFM_per_Q	1.11 (1.08, 1.13)	< 0.001	1.37 (1.32, 1.43)	< 0.001	1.33 (1.26, 1.41)	< 0.001
Quartiles of RFM						
Q1	Ref		Ref		Ref	
Q2	1.09 (1.05, 1.14)	< 0.001	1.12 (1.07, 1.16)	< 0.001	1.11 (1.05, 1.17)	< 0.001
Q3	1.04 (1.00, 1.08)	0.061	1.31 (1.24, 1.39)	< 0.001	1.27 (1.18, 1.37)	< 0.001
Q4	1.20 (1.15, 1.25)	< 0.001	1.60 (1.50, 1.71)	< 0.001	1.55 (1.42, 1.70)	< 0.001
*p* for trend	< 0.001		< 0.001		< 0.001	

*Note:* Model 1: no adjustment for any potential influencing factors. Model 2: adjusted for age and gender. Model 3: adjusted for age, gender, marital status, education, smoking, and drinking.

Furthermore, studies have shown that there is a positive correlation between RFM levels and the risk of CMM (Tables 3 and 4). In the CHARLS database, after adjusting for potential confounding variables (Model 3), compared with Q1, the risk of CMM increases with the elevation of RFM, and the ORs for Q2, Q3, and Q4 are 1.12 (1.10, 1.15), 1.19 (1.15, 1.24), and 1.34 (1.29, 1.39), respectively (Table 3). For participants in NHANES, in Model 3, for every 1‐unit increase in RFM, the risk of CMM will increase by 33% (OR = 1.33, 95% CI: 1.26–1.41) (Table 4).

### 3.3. RCS Analysis

To deeply investigate the relationships between SCR/CysC, RFM, and CMM, through RCS analysis, the results showed that there was a significant nonlinear relationship (*p* < 0.001) between SCR/CysC (Figure 2a) or RFM (Figure 2c) and CMM in CHARLS. In NHANES, the results also revealed the nonlinear correlations among SCR/CysC (Figure 3a), RFM (Figure 3), and CMM (*p* < 0.001).

Figure 2Restricted cubic spline curve for the relationships among SCR/CysC, RFM, and CMM in the CHARLS. RCS curves of the incidence CMM for the (a) SCR/CysC, (b) SCR/CysC + gender, (c) RFM, and (d) RFM + gender groups.

**Figure:**
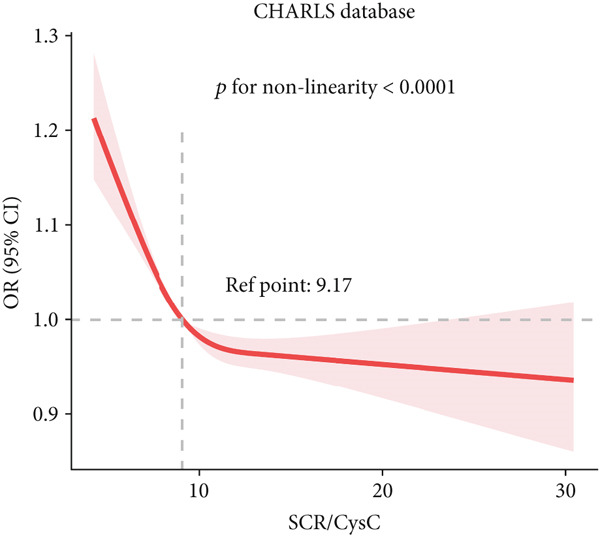
(a)

**Figure:**
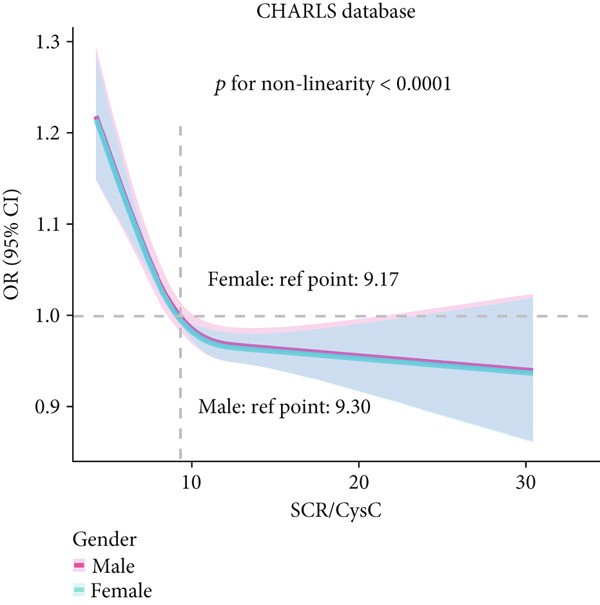
(b)

**Figure:**
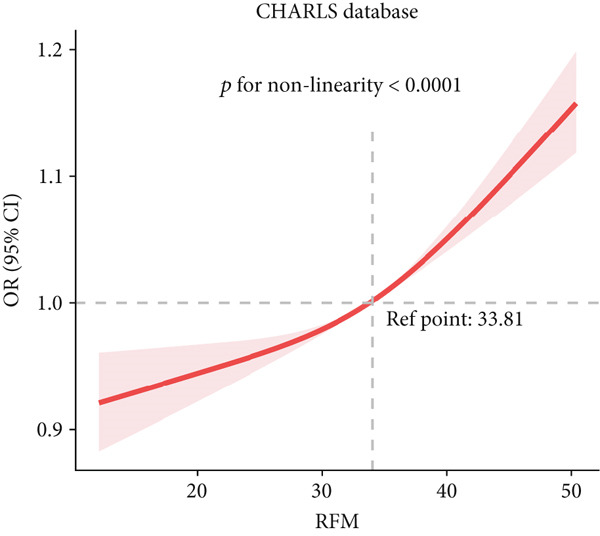
(c)

**Figure:**
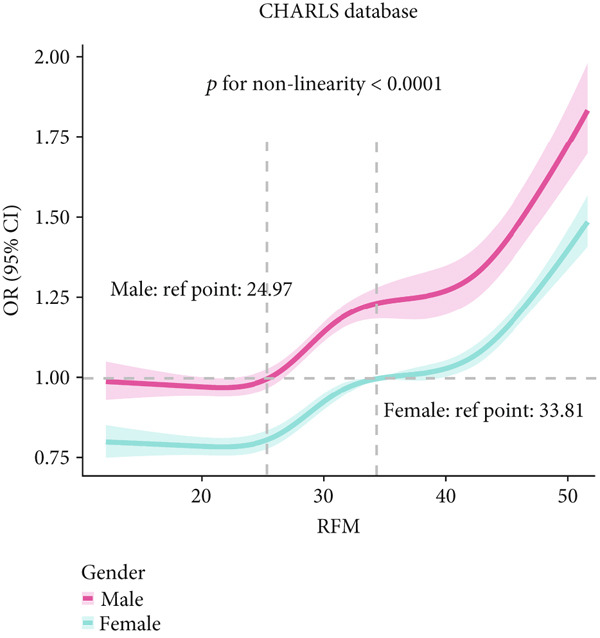
(d)

Figure 3Restricted cubic spline curve for the relationships among SCR/CysC, RFM, and CMM in NHANES. RCS curves of the incidence of CMM for the (a) SCR/CysC, (b) SCR/CysC + gender, (c) RFM, and (d) RFM + gender groups.

**Figure:**
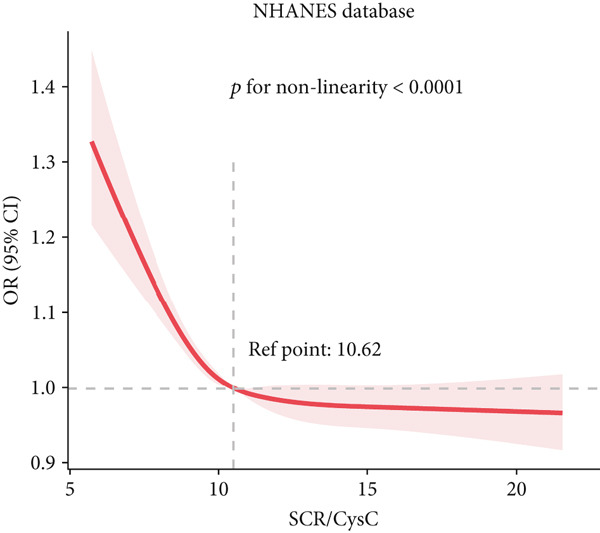
(a)

**Figure:**
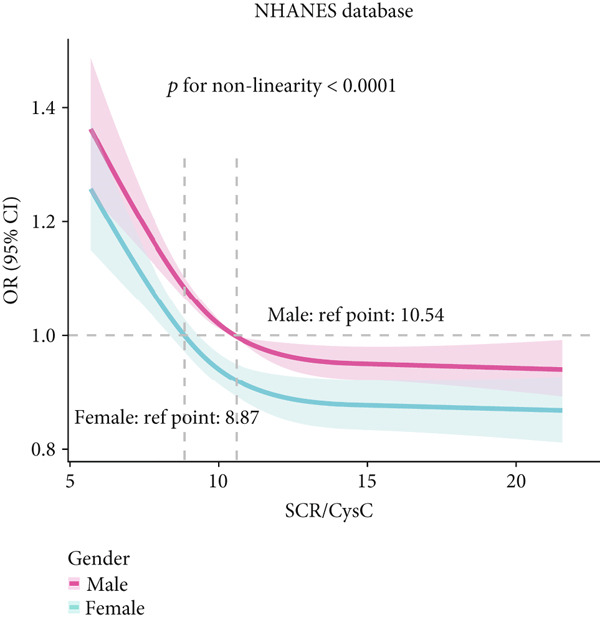
(b)

**Figure:**
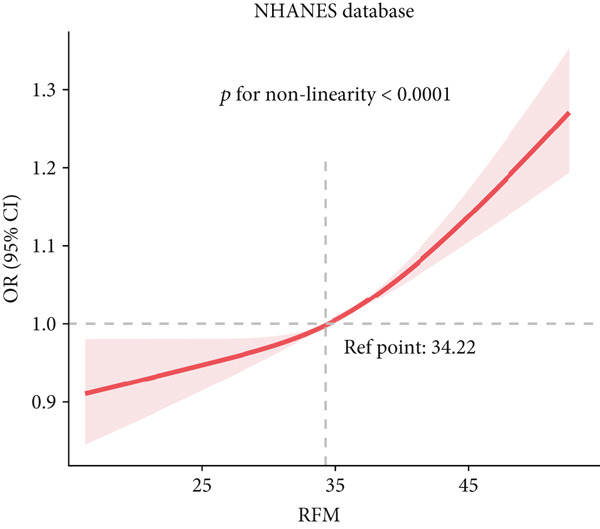
(c)

**Figure:**
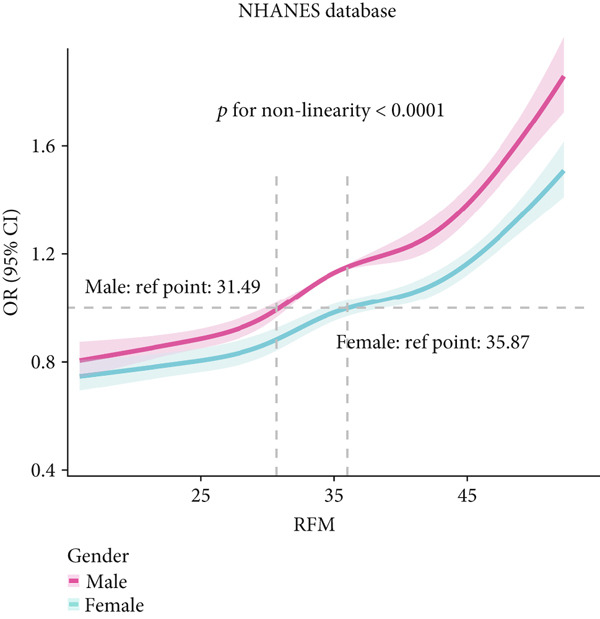
(d)

In the RCS analysis, when the OR value is 1, it indicates that the effect of the factor on the outcome is not significant. Among them, when OR = 1, the SCR/CysC value of NHANES participants was 10.62 (Figure 3a), and that of CHARLS participants was 9.17 (Figure 2a). This indicates that under the same risk threshold, the SCR/CysC level of the Chinese population is relatively low. It is worth noting that in both the NHANES and CHARLS studies, the SCR/CysC values of male participants were generally higher than those of female participants (Figures 2b and 3b).

For RFM, when OR = 1, the RFM value of the NHANES participants was 34.22 (Figure 3c), and that of the CHARLS participants was 33.81 (Figure 2c). This further indicates that, at the same risk level, the exposure level of RFM among the Chinese population is also relatively low. In addition, this study also found that the RFM values of male participants in NHANES and CHARLS were usually lower than those of female participants (Figures 2d and 3d).

### 3.4. Subgroup Analysis

All participants of CHARLS and NHANES were divided into those aged 60 and above and those under 60 for analysis during the interaction analysis (Figures 4 and 5). In CHARLS, marital status (*p* = 0.012) and education level (*p* = 0.017) both showed significant interactions with SCR/CysC: among unmarried individuals and those with a high school education or above, the association between a decrease in SCR/CysC and the risk of CMM was stronger (Figure 4a), while the association strength between RFM and CMM was significantly enhanced in individuals under 60 years old and smokers (*p* interaction < 0.01) (Figure 4b).

Figure 4Subgroup analysis for the associations between SCR/CysC, RFM, and CMM in CHARLS. (a) Subgroup analysis of the association between SCR/CysC and CMM. (b) Subgroup analysis of the association between RFM and CMM.

**Figure:**
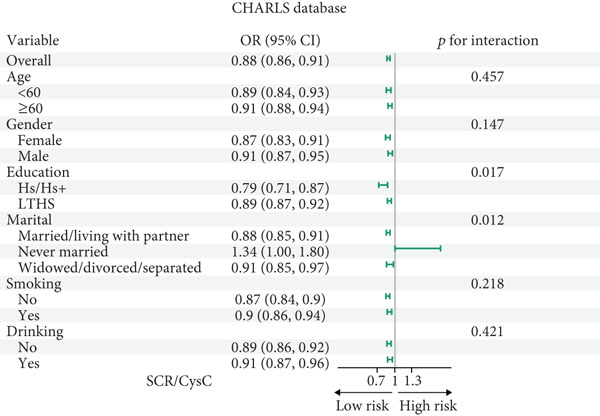
(a)

**Figure:**
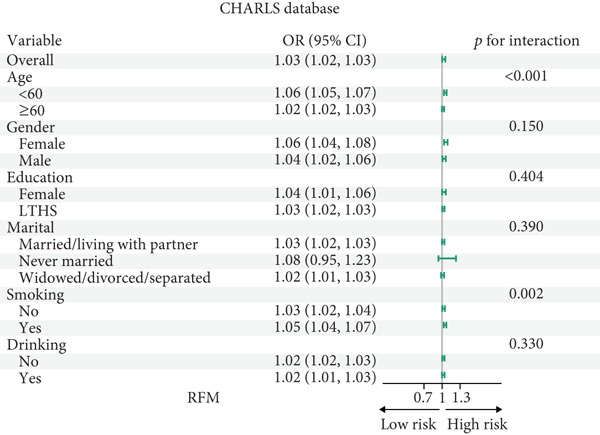
(b)

Figure 5Subgroup analysis of the associations among SCR/CysC, RFM, and CMM in NHANES. (a) Subgroup analysis of the association between SCR/CysC and CMM. (b) Subgroup analysis of the association between RFM and CMM.

**Figure:**
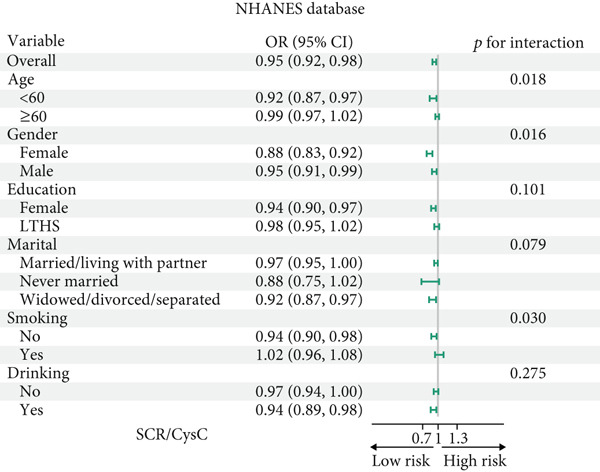
(a)

**Figure:**
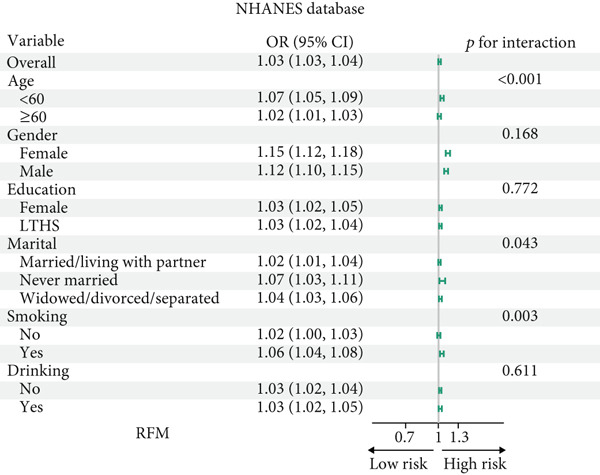
(b)

In NHANES, age (*p* = 0.018), gender (*p* = 0.016), and smoking status (*p* = 0.030) all showed significant interactions with SCR/CysC: Among individuals under 60 years old, females, and smokers, the association between a decrease in SCR/CysC and the risk of CMM was stronger (Figure 5a), while the association strength between RFM and CMM was significantly enhanced in individuals under 60 years old, unmarried/dwelling alone, and smokers (*p* interaction < 0.05) (Figure 5b).

### 3.5. Survival Analysis of NHANES Participants

Kaplan–Meier curve analysis showed that during the 12‐year follow‐up period, for CMM participants, reduced SCR/CysC levels are associated with increased mortality risk in CMM patients (log‐rank *p* < 0.001) (Figure 6a). However, for RFM, as the interquartile increased (from Q1 to Q4), the survival rate did not show the positive trend as expected. It is worth noting that in the Q2 quantile of RFM, the survival rate of the participants was relatively low (Figure 6b).

Figure 6Kaplan–Meier survival curves from NHANES. (a) Kaplan–Meier survival curve for CMM patients at different SCR/CysC levels. (b) Kaplan–Meier survival curves for CMM patients at different RFM levels.

**Figure:**
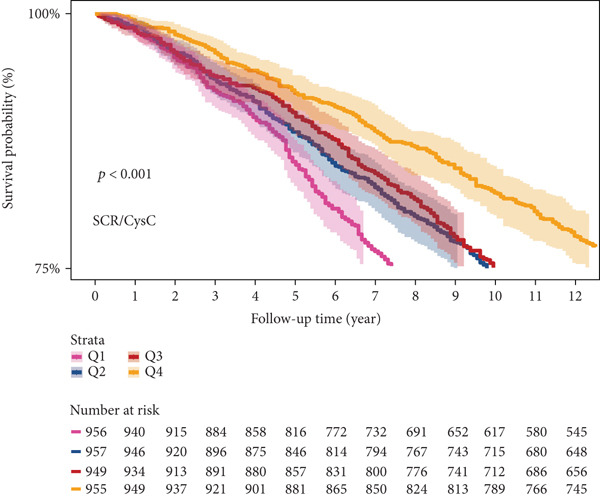
(a)

**Figure:**
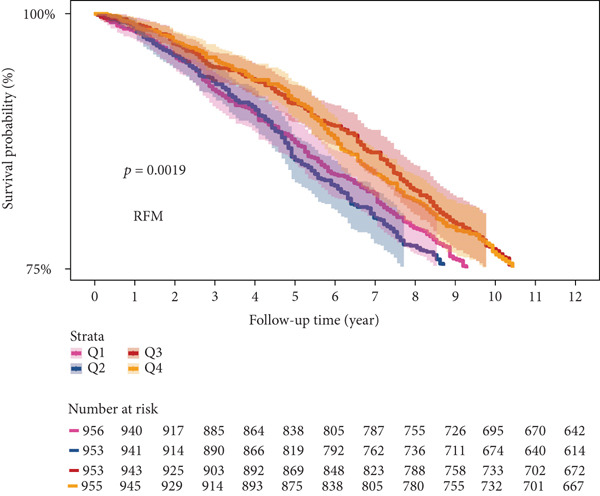
(b)

## 4. Discussion

This study, based on the analysis of the CHARLS and NHANES databases, has found that SCR/CysC and RFM can serve as potential indicators for the assessment of CMM. SCR/CysC levels were found to be negatively correlated with the risk of developing CMM, whereas RFM levels were positively related to the risk of developing CMM. In addition, at the risk threshold of OR = 1, the exposure–response inflection points for both SCR/CysC and RFM were observed to be lower in the Chinese participants than in the US participants. Notably, SCR/CysC levels were generally higher in men than in women, whereas RFM values were lower in men than in women.

RFM, a new index of adiposity [23], demonstrates a robust correlation with multiple health concerns. Specifically, elevated RFM is not only closely related to metabolic abnormalities but also significantly associated with an increased incidence of CVD [14, 24]. When the RFM thresholds of men and women exceed 30 and 45, respectively, the risk of cardiovascular death increases significantly [12]. This discovery further reinforces the importance of RFM in assessing the risk of CVD. Furthermore, Peng et al. predicted the incidence of hypertension through RFM and found that the optimal threshold for men was 24.67 and for women was 35.73. Beyond this threshold, the risk of developing hypertension increased [10]. Previous studies have also shown that elevated RFM may indicate an increased susceptibility of individuals to diseases such as diabetes and fatty liver [11, 19, 24]. Therefore, this study delves deeply into the relationship between RFM and CMM and finds a significant association between elevated RFM and increased risk of CMM. This finding not only aligns with previous studies on the detrimental effects of RFM on metabolic and cardiovascular health but also offers new perspectives on understanding and assessing the pathogenesis of CMM.

Compared with the central obesity indicator WHtR, RFM has certain advantages. By incorporating sex differences and combining the ratio of height to WC, it can more comprehensively reflect the distribution of body fat, especially having more advantages in distinguishing between subcutaneous fat and visceral fat. Elevated RFM usually reflects an increase in body fat content, especially the accumulation of visceral fat. Visceral adipose tissue is not only an energy storage organ but also has active endocrine functions and is capable of secreting various bioactive substances, such as proinflammatory cytokines (for example, TNF‐*α* and IL‐6) [25]. These substances can trigger chronic inflammatory responses, and chronic inflammation is one of the important links in the occurrence and development of CMM [26]. Inflammatory factors can damage vascular endothelial cells, leading to vascular endothelial dysfunction, thereby promoting the formation of atherosclerosis and increasing the risk of CVDs [27]. Meanwhile, these inflammatory factors can also interfere with the insulin signaling pathway, leading to insulin resistance and thereby causing a series of metabolic disorders such as elevated blood sugar and dyslipidemia, further aggravating the CMM [28].

The SCR/CysC ratio has been widely recognized as a key biomarker for assessing muscle mass and predicting the risk of sarcopenia [29]. The increase of this ratio not only indicates an increase in muscle mass, but also, studies have shown that an individual′s ability to maintain glucose homeostasis under normal blood sugar and high insulin conditions is significantly enhanced, which is of great significance for the prevention of diabetes and its related complications [30]. Conversely, when the SCR/CysC ratio decreases, it often indicates the presence of sarcopenia, and this phenomenon is closely related to a variety of adverse health outcomes, including CVDs, osteoporosis, and a significant decline in overall quality of life [21, 31, 32]. Prior research has revealed the relationships between the SCR/CysC ratio and a variety of health indicators. Beyond its inverse correlation with blood pressure and HbA1c [33], a reduced SCR/CysC ratio is linked to an elevated risk of CVD, worsening metabolic syndrome, and cognitive decline [34, 35]. Based on this, this study deeply explored the relationship between the SCR/CysC ratio and the progression of CMM. The research results show that there is a significant negative correlation between the two; that is, a higher SCR/CysC ratio is closely related to a significant reduction in the risk of individual CMM. These findings not only further expand our understanding of the mechanism of action of SCR/CysC biomarkers but also provide a solid scientific basis for constructing an early warning system and personalized intervention strategy aimed at reducing the risk of individual CMM.

Based on mortality data from the NHANES database, both the RFM and SCR/CysC metrics were analyzed for their impact on the survival of CMM patients. The research results indicate that higher levels of SCR/CysC may be associated with a reduced risk of death in patients with CMM, which is consistent with the results of the correlation analysis. On the other hand, while the correlation analysis between RFM and the risk of CMM incidence revealed a positive relationship, the survival analysis yielded unexpected findings. Instead of showing a positive correlation between CMM mortality and RFM levels as expected, higher RFM levels were associated with increased mortality, peaking at the Q2 quantiles. This might be a local statistical phenomenon caused by the discrete grouping.

## 5. Strengths and Limitations

Leveraging pooled data from two nationally representative cohorts (CHARLS and NHANES), this study achieves high external validity and statistical robustness through a large analytic sample and multiple models. Nevertheless, the cross‐sectional design precludes causal inference, and the ascertainment of CMM relied on self‐reported physician diagnosis, introducing possible information bias. Although participants were excluded only for missing values on key exposure (SCR, CysC, and RFM) or outcome (CMM) variables, baseline characteristics differed between included and excluded individuals, potentially limiting generalizability. Furthermore, the CHARLS data only has complete biochemical indicators and medication data for approximately 50% of the participants. If only the samples with complete data are included, it will significantly reduce the sample size and may introduce selection bias.

## 6. Conclusion

This study demonstrates that SCR/CysC and RFM serve as reliable indicators of muscle mass and relative fat content, respectively. Higher SCR/CysC ratios and lower RFM levels correlate with reduced CMM risk.

**Nomenclature**
BMI: body mass index
CHARLS: Chinese Health and Retirement Longitudinal Study
CI: confidence interval
CMM: cardiometabolic multimorbidity
CVD: cardiovascular disease
DBP: diastolic blood pressure
FBG: fasting blood glucose
HbA1c: glycated hemoglobin
HS/HS+: high school or above
LTHS: below high school
NHANES: National Health and Nutrition Examination Survey
OR: odds ratio
RCS: restricted cubic spline
RFM: relative fat mass
SBP: systolic blood pressure
SCR/CysC: serum creatinine–cystatin C ratio
TyG: triglyceride–glucose
WC: waist circumference
WHtR: waist‐to‐height ratio

## Ethics Statement

The CHARLS was ethically approved by the institutional review board at Peking University (IRB00001052‐11015). NHANES received ethical approval from the NCHS Research Ethics Review Board (ERB). All participants signed an informed consent form before taking part in the survey.

## Conflicts of Interest

The authors declare no conflicts of interest.

## Funding

No funding was received for this manuscript.

## Supporting information


**Supporting Information** Additional supporting information can be found online in the Supporting Information section. Table S1. Baseline characteristics between observations included and excluded based on CHARLS and NHANES.

## Data Availability

The datasets utilized in this study are sourced from two reputable and publicly accessible resources: CHARLS, accessible at http://charls.pku.edu.cn/, and NHANES, accessible at https://www.cdc.gov/nchs/nhanes/index.htm. The datasets analyzed during the current study are available from the corresponding authors on reasonable request.
