# Supplementary material for: Association Between Relative Fat Mass and the Serum Creatinine/Cystatin C Ratio and Cardiometabolic Multimorbidity: Evidence From Two Large Population‐Based Surveys
Source: J Diabetes Res. 2025 Dec 22;2025:4075738. doi: 10.1155/jdr/4075738 (PMC12767230; doi:10.1155/jdr/4075738)
Supplement: Supplementary file 1 — Supporting Information Additional supporting information can be found online in the Supporting Information section. Table S1. Baseline characteristics between observations included and excluded based on CHARLS and NHANES. [file JDR-2025-4075738-s001.docx]

**Supplementary materials**

**Table S1. Baseline characteristics between observations included and excluded based on CHARLS and NHANES.**

| **Characteristic** | **CHARLS** | | | **NHANES** | | |
| --- | --- | --- | --- | --- | --- | --- |
|  | **Excluded** | **Included** | ***P*** | **Excluded** | **Included** | ***P*** |
| **N** | 11803 | 9292 |  | 27305 | 3821 |  |
| **Age**  **(mean ± SD)** | 57.08(11.5) | 62.40(8.9) | <0.001 | 24.86(22.5) | 64.05(11.5) | <0.001 |
| **Gender (%)** |  |  | 0.015 |  |  | 0.011 |
| Female | 6085(51.7) | 4959(53.4) |  | 14059(51.5) | 1883 (49.3) |  |
| Male | 5692(48.3) | 4333(46.6) |  | 13246(48.5) | 1938(50.7) |  |
| **Marital (%)** |  |  | <0.001 |  |  | <0.001 |
| Married/Living with Partner | 10351(87.7) | 7967(85.7) |  | 6799(41.5) | 2402(64.9) |  |
| Never Married | 129(1.1) | 63(0.7) |  | 7254(44.3) | 174(4.7) |  |
| Widowed/Divorced/Separated | 1323(11.2) | 1262(13.6) |  | 2340(14.3) | 1126(30.4) |  |
| **Education (%)** |  |  | <0.001 |  |  | <0.001 |
| HS/HS+ | 1105(15.0) | 924(10.0) |  | 7859(68.3) | 2364(61.9) |  |
| LTHS | 6271(85.0) | 8361(90.0) |  | 3647(31.7) | 1456(38.1) |  |
| **Smoking (%)** |  |  | 0.114 |  |  | <0.001 |
| No | 4674(58.9) | 5358(57.7) |  | 2671(50.3) | 1418(68.1) |  |
| Yes | 3262(41.1) | 3929(42.3) |  | 2637(49.7) | 664(31.9) |  |
| **Drinking (%)** |  |  | <0.001 |  |  | <0.001 |
| No | 7389(63.5) | 6117(65.9) |  | 6446(68.7) | 2401(64.8) |  |
| Yes | 4241(36.5) | 3165(34.1) |  | 2936(31.3) | 1306(35.2) |  |
| **RFM**  **(mean (SD))** | 31.58(44.1) | 31.88(14.7) | 0.549 | 31.19(9.3) | 36.30(7.9) | <0.001 |
| **SCR/CysC (mean (SD))** | 10.05(2.6) | 9.51(2.7) | <0.001 | 11.16(2.9) | 10.88(3.4) | <0.001 |
| **Hypertension (%)** |  |  | <0.001 |  |  | <0.001 |
| No | 4741(60.8) | 5276(56.8) |  | 15136(79.2) | 1412(37.0) |  |
| Yes | 3052(39.2) | 4016(43.2) |  | 3987(20.8) | 2409(63.0) |  |
| **Diabetes (%)** |  |  | <0.001 |  |  | <0.001 |
| No | 3586(80.6) | 7785(83.8) |  | 6186(89.1) | 2541(66.5) |  |
| Yes | 865(19.4) | 1507(16.2) |  | 758(10.9) | 1280(33.5) |  |
| **CMM (%)** |  |  | <0.001 |  |  | <0.001 |
| No | 314(56.4) | 7759(83.5) |  | 6098(92.0) | 2539(66.4) |  |
| Yes | 243(43.6) | 1533(16.5) |  | 533(8.0) | 1282(33.6) |  |

Note: The mean SD was used for continuous variables, and the percentage (%) was used for categorical variables. HS/HS+: educational level of high school and above, LTHS: educational level below upper secondary. RFM: relative fat mass. SCR/CysC: serum creatinine / Cystatin C.
